# NT-proBNP, C-Reactive Protein and Soluble uPAR in a Bi-Ethnic Male Population: The SAfrEIC Study

**DOI:** 10.1371/journal.pone.0058506

**Published:** 2013-03-13

**Authors:** Ruan Kruger, Rudolph Schutte, Hugo W. Huisman, Peter Hindersson, Michael H. Olsen, Jesper Eugen-Olsen, Aletta E. Schutte

**Affiliations:** 1 Hypertension in Africa Research Team (HART); Department of Physiology, North-West University, Potchefstroom, North-West Province, South Africa; 2 Department of Biochemistry, Hospital Vendsyssel, Hjørring, Denmark; 3 Cardiovascular Prevention Clinic, Department of Endocrinology, Odense University Hospital, University of Southern Denmark, Odense, Denmark; 4 Clinical Research Centre 136, Copenhagen University, Hvidovre Hospital, Hvidovre, Denmark; University of South Florida, United States of America

## Abstract

**Objective and design:**

This cross-sectional study aimed to investigate associations between a marker of cardiac strain, the N-terminal prohormone B-type natriuretic peptide (NT-proBNP), and inflammation as reflected by either a conventional or novel inflammatory marker in a bi-ethnic South African cohort.

**Methods and subjects:**

We measured NT-proBNP, C-reactive protein (CRP) and plasma-soluble urokinase plasminogen activator receptor (suPAR) levels along with conventional biomarkers in black (n = 117) and white (n = 116) men.

**Results:**

NT-proBNP, CRP and suPAR levels were higher in black compared to white men. NT-proBNP was significantly associated with both CRP (r = 0.38; p = 0.001) and suPAR (r = 0.42; p<0.001) in black men only. After full adjustment in multiple regression analyses, the above associations of NT-proBNP with CRP (β = 0.199; p = 0.018) and suPAR (β = 0.257; p<0.01) were confirmed in black men.

**Conclusion:**

These results suggest that a low-grade inflammatory state as reflected by both a conventional and novel marker of inflammation may contribute to higher cardiovascular risk as reflected by the associations obtained with a marker of cardiac strain in black South African men.

## Introduction

High morbidity and mortality among the black population in South Africa is a major concern due to the high prevalence of communicable and non-communicable diseases, especially in communities of low socio-economic standing with limited healthcare facilities [1]. A low-grade inflammatory state, heightened by lifestyle and environmental factors, is believed to underlie the elevating adverse health outcomes observed in black patients [2]. The biological effects of pro-inflammatory cytokines and other components of inflammation are augmented by acute or chronic cardiovascular insults [3]. Pro-inflammatory cytokines, acute-phase reactants as well as cell adhesion and signaling molecules such as plasma-soluble urokinase plasminogen activator receptor (suPAR) and C-reactive protein (CRP) seem to play a role in mediating low-grade inflammation [3].

CRP, which reflects systemic inflammation, is elevated in acute heart failure and N-terminal prohormone B-type natriuretic peptide (NT-proBNP), reflecting cardiac strain, are proposed reliable risk markers of cardiovascular disease as well as good predictors of cardiovascular morbidity and mortality [4], [5], [6]. In addition, a novel marker of inflammation and subclinical atherosclerosis (suPAR) is associated with the risk of developing cardiovascular disease [7], and is also suggested to be an independent marker of subclinical organ damage and increased cardiovascular risk [8].

The associations of NT-proBNP with markers of inflammation have been studied in part in white populations of American and European descent [7], [9], [10], [11], but little is known about these associations in black populations. The aim of this study was therefore to investigate whether subclinical cardiovascular damage assessed by NT-proBNP is associated with low-grade inflammation as reflected by a conventional and novel inflammatory marker.

## Methods

### Study population

#### Ethics Statement

The SAfrEIC study was approved (06M01) by the Ethics Review Board of the North-West University and the study protocol conformed to the ethical guidelines of the Declaration of Helsinki (2008) for investigation of human participants. This cross-sectional study formed part of a larger South African investigation on the role of Sex, Age and Ethnicity on Insulin sensitivity and Cardiovascular function (SAfrEIC) study that involved 382 black and 372 white participants from the North West province of Southern Africa. This sub-study included 117 black and 116 white men after excluding all patients who were using antihypertensive or anti-inflammatory medication (n = 41) and those infected with HIV (n = 55).

### Clinical procedures

Approximately 10 to 20 recruited participants visited the Metabolic Unit facility daily at the Potchefstroom campus of the North-West University over a period of seven weeks. Each participant gave written informed consent to take part in the study after all the procedures were comprehensively explained. A participant sheet guided the participants through the different research stations where various measurements were performed. Basic health and demographic questionnaires were completed during the morning. An informative description regarding the results of the health assessment was given to each participant at the end of the study. In the event where abnormalities became evident (e.g. hypertension or diabetes), the participant was advised to visit their local clinic, hospital or physician.

### Cardiovascular measurements

Duplicate office blood pressure measurements were taken with the Omron HEM-757 (Omron, Kyoto, Japan) apparatus. The first blood pressure measurement was taken after an initial 10 minute resting period and a second measurement was taken 5 minutes after the first. Pulse pressure was subsequently calculated. Participants with a systolic (SBP) ≥140 mmHg and/or diastolic blood pressure (DBP) ≥90 mmHg were considered hypertensive [12]. Heart rate and Windkessel arterial compliance were determined with the Finometer apparatus (FMS, Finapres Measurement Systems, Amsterdam, the Netherlands) [13], [14]. The pulse wave velocity (PWV), as arterial stiffness marker, was measured with the Complior SP Acquisition system (Artech-Medical, Pantin, France).

### Anthropometric measurements

Body height was measured to the nearest 1.0 cm by using the Invicta Stadiometer (Invicta Plastics 1465, London, UK) and body weight to the nearest 0.1 kg (Precision Health Scale, A & D Company, Japan). Subsequently, the body mass index (BMI) was calculated for each participant. The waist circumference was measured at the midpoint between the lowest rib and the top of the iliac crest with a Holtain non-stretchable, flexible metal measuring tape [15].

### Biochemical measurements

Participants were requested to fast for a minimum of eight hours. Fasting lipids (total cholesterol (TC), high-density lipoprotein cholesterol (HDL), and triglycerides (TG)), serum glucose, γ-glutamyl transferase, creatinine and high-sensitivity serum CRP were determined with the Konelab autoanalyzer (Thermo Fisher Scientific, Vantaa, Finland). Low density lipoprotein cholesterol (LDL) was determined with the Friedewald formula: LDL = TC – HDL – TG/2.17. Insulin was determined with the ST AIA-PACK IRI kit (TOSOH AIA, Inc., Toyama, Japan; catalogue no. 025260) using a two-site immuno-enzymometric assay. Serum cotinine was determined with the IMMULITE 2000 nicotine metabolite assay (Siemens Medical Solutions Diagnostics, Los Angeles, CA). The Elecsys proBNP sandwich immunoassay was used on an Elecsys 2010 (Roche Diagnostics, Mannheim, Germany) to determine the serum NT-proBNP concentration of each participant. Plasma (EDTA) suPAR levels were measured using the suPARnostic® ELISA kit (ViroGates, Copenhagen, Denmark). Human immunodeficiency virus status was determined directly after blood sampling with rapid tests according to the protocol of the National Department of Health of South Africa. Serum was used for testing with the First Response Test (PMC Medical, India) and was repeated with the Pareeshak test (BHAT Bio-tech India) to confirm the results.

### Statistical analyses

Statistica software v10.0 (StatSoft, Inc., Tulsa, OK, USA) was used for database management and statistical analyses. The normal distribution of the variables was tested prior to any further statistical analyses. Variables that deviated from normality (NT-proBNP, CRP, suPAR, γ-glutamyl transferase, blood glucose and cotinine) were logarithmically transformed. Associations of NT-proBNP with markers of inflammation were tested for interaction with ethnicity by performing the appropriate interaction terms. Chi-square tests (χ^2^) were used to compare proportions and independent t-tests to compare the means of continuous variables. We plotted unadjusted associations between NT-proBNP and markers of inflammation in single regression analysis. We divided CRP and suPAR values into quartiles to explore associations with NT-proBNP levels, while adjusting for SBP and arterial compliance in analysis of covariance (ANCOVA). A standard multiple regression analyses were performed to investigate independent associations between NT-proBNP and inflammatory markers. Several covariates were considered for entry into the regression model including age, BMI, SBP, arterial compliance, serum creatinine, γ-glutamyl transferase, fasting glucose, total cholesterol to high density lipoprotein cholesterol ratio and cotinine. Probability values of α≤0.05 were considered statistically significant.

## Results

Detailed clinical and basic population characteristics are presented in Table 1. The total population was stratified by ethnicity, due to significant interactions on the main effects of ethnicity on the associations of NT-proBNP with CRP (F(233) = 27.02; p<0.0001) and suPAR (F(233) = 30.3; p<0.0001). The black men were older (p = 0.003) and smoked more compared to the white men (p<0.0001). This was also supported by the higher cotinine levels (p<0.0001). The self-reported use of alcohol (82.2% vs. 76.3%; p = 0.26) was similar, but the levels of γ-glutamyl transferase was higher in the black men (p<0.0001). Metabolic risk factors including BMI, serum glucose and lipid levels were lower in the black men. Mean levels of NT-proBNP (p<0.0001), CRP (p = 0.038) and suPAR (p<0.0001) were higher in the black compared to white men.

**Table 1:** Clinical and basic population characteristics of the bi-ethnic male cohort.

	African	Caucasians	p–value
	n = 117	n = 116	
Age, years	41.4±13.8	36.3±11.7	0.003
Body mass index, kg/m^2^	20.4±4.2	27.8±5.0	<0.0001
***Biochemical analyses***			
NT-proBNP, ng/ml	29.6 (5.5–187.8)	10.9 (5.1–44.7)	<0.0001
C-reactive protein, mg/l	1.73 (0.007–22.86)	1.02 (0.007–9.22)	0.038
Soluble uPAR, ng/ml	2.95 (1.66–5.00)	2.02 (1.32–3.21)	<0.0001
Serum creatinine, µmol/l	63.9±10.2	71.4±10.5	<0.0001
Serum glucose, mmol/l	4.97 (4.03–6.42)	5.54 (4.59–7.13)	<0.0001
TC:HDL, mmol/l	2.88±1.08	5.24±2.07	<0.0001
Triglycerides, mmol/l	1.07±0.55	1.58±0.97	<0.0001
LDL, mmol/l	2.22±0.89	3.86±1.37	<0.0001
Insulin, µU/ml	4.94±5.17	10.1±9.1	<0.0001
***Cardiovascular measurements***			
Systolic blood pressure, mmHg	130.4±20.6	121.7±11.0	<0.0001
Diastolic blood pressure, mmHg	84.4±13.7	77.6±8.1	<0.0001
Pulse pressure, mmHg	46.1±11.5	44.1±8.5	0.13
Heart rate, bpm	67.9±14.5	66.1±9.1	0.25
Arterial compliance, ml/mmHg	1.67±0.51	2.53±0.50	<0.0001
Hypertension status, n (%)	44 (37.6)	8 (6.8)	<0.0001
***Lifestyle***			
Smoking, n (%)	87 (74.4)	25 (21.2)	<0.0001
Cotinine, ng/ml	115.9 (9.0–500.0)	18.3 (9.0–371.0)	<0.0001
Alcohol use, n (%)	97 (82.2)	90 (76.3)	0.26
γ-Glutamyl transferase, U/l	79.5 (20.7–487.2)	35.5 (17.7–101.2)	<0.0001

Values are arithmetic mean ± SD, geometric mean (5^th^ and 95^th^ percentiles) or number of participants. *Abbreviations:* TC:HDL – total cholesterol to high density lipoprotein cholesterol ratio; uPAR – urokinase plasminogen activator receptor.

**Figure 1:**
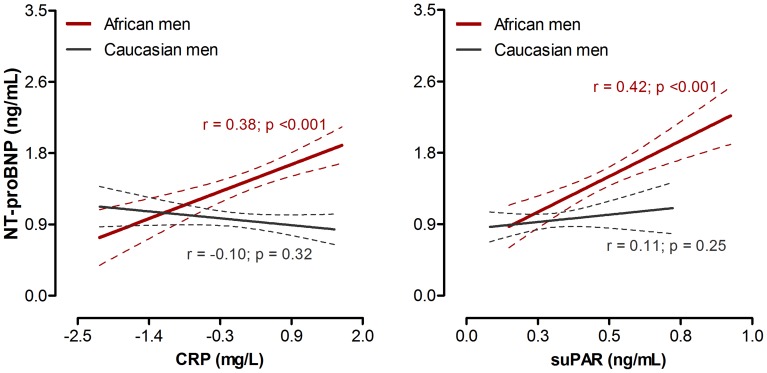
Single regression analyses of NT-proBNP with markers of inflammation in black and white men.

**Figure 2:**
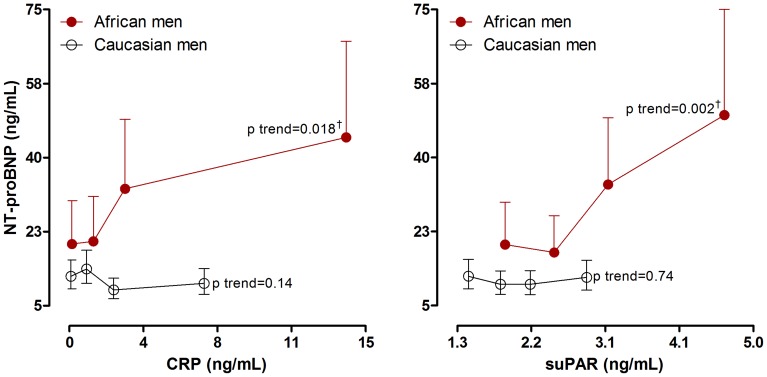
NT-proBNP levels by quartiles of CRP and suPAR, adjusted for systolic blood pressure and arterial compliance. Probability denoted significance for trend; †p<0.01 (Q_1_ vs. Q_4_).

In single regression analysis (Figure 1), NT-proBNP correlated positively with both CRP (r = 0.38; p<0.001) and suPAR (r = 0.42; p<0.001) in black men only. By plotting NT-proBNP by quartiles of CRP and suPAR in both black and white men (Figure 2), with adjustments for SBP and arterial compliance, the relationships above were supported further in the black men (CRP; p for trend = 0.018 and suPAR; p for trend = 0.002). Again, no associations were observed in the white men (CRP; p for trend = 0.14 and suPAR; p for trend = 0.74).

The independent associations between NT-proBNP and inflammatory markers are shown in Table 2. After adjusting for age, BMI, SBP, arterial compliance, serum creatinine and γ-glutamyl transferase (variables that entered the model), the positive associations with CRP (p = 0.018) and suPAR (p = 0.007) were confirmed and remained non-significant in the white men (CRP; β = −0.017, p = 0.86 and suPAR; β = 0.121, p = 0.23).

**Table 2:** Multiple regression analysis of NT-proBNP with CRP and suPAR in African men.

	CRP, mg/l	suPAR, ng/ml
*R^2^*	0.43	0.44
*Adjusted R^2^*	0.39	0.41
***Standard β (95% CI)***		
NT-proBNP, ng/ml	0.199 (0.042 to 0.356)*	0.257 (0.088 to 0.425)†
Age, years	0.559 (0.279 to 0.839)‡	0.515 (0.242 to 0.788)‡
Body mass index, kg/m^2^	−0.123 (−0.298 to 0.053)	−0.142 (−0.311 to 0.026)
Systolic blood pressure, mmHg	0.393 (0.188 to 0.497)‡	0.393 (0.193 to 0.592)‡
Arterial compliance, ml/mmHg	0.276 (−0.057 to 0.608)	0.222 (−0.101 to 0.545)
Serum creatinine, µmol/l	−0.191 (−0.349 to −0.033)*	−0.145 (−0.305 to 0.015)
γ-Glutamyl transferase, U/l	−0.096 (−0.264 to 0.072)	−0.185 (−0.357 to −0.013)*

Superscript symbol denotes significance for:

* p≤0.05;

† p≤0.01;

‡ p≤0.001.

*Abbreviations:* CI – confidence intervals; TC:HDL – total cholesterol to high density lipoprotein cholesterol ratio.

CRP and suPAR levels were also compared after adjusting for BMI, since the BMI differed largely between these groups. After this adjustment, the initial difference between black and white men remained for both CRP (2.32 mg/l vs. 0.73 mg/l; p<0.001), and suPAR (2.97 ng/ml vs. 1.99 ng/ml; p<0.001). It is also noteworthy to mention that the mean blood pressures of the black men were high normal compared to white men with optimal pressures. By performing ANCOVA with adjustments for BMI, SBP, cotinine and γ-glutamyl transferase the initial difference in CRP levels between black and white men disappeared (1.68 mg/l vs. 1.01 mg/l; p = 0.17). None of these variables seem to alter the difference in mean suPAR levels in black or white men (2.67 ng/ml vs. 2.20 ng/ml; p<0.01).

## Discussion

This study investigated the associations of NT-proBNP with CRP and suPAR in black and white South African men. NT-proBNP associated positively with both inflammatory markers in black men. However, this relationship was absent in the white group. It is known that CRP reflects metabolic inflammation with its relation to BMI and lipids [16], while suPAR may be more related to cell-specific immune activation of the circulation [17]. Our results confirm the conclusion of a previous study by Jensen *et al.* that suggested a positive relationship between NT-proBNP and inflammatory markers including CRP, orosomucoid, haptoglobulin and alpha1-antitrypsin in European (Swedish) men and woman [11]. No study investigated the relationship between NT-proBNP and suPAR; however Eugen-Olsen *et al.* investigated the potential of suPAR as a marker of adverse cardiovascular outcome in over 2500 Danish men and women. From those results, suPAR associated with an increased risk of cardiovascular disease, cancer, type 2 diabetes and mortality, more strongly in men than in women [7]. Our study is the first to investigate NT-proBNP, CRP and suPAR in a bi-ethnic cohort from South Africa. Therefore, our results suggest that a potential low-grade inflammatory state in black men may predispose them to premature development of subclinical cardiac strain and future cardiovascular events.

Increased levels of the cardiac hemodynamic volume load biomarker, NT-proBNP, reflect the risk of developing left ventricular dysfunction and congestive heart failure [18]. In addition, elevated CRP and suPAR levels indicate active systemic as well as low-grade inflammation, especially in atherosclerotic progression [19], [20]. Therefore, the associations of NT-proBNP with both these markers in black men suggest the link between the potential developments of subclinical cardiac damage in unison with early onset atherosclerosis. This relationship might also explain the higher systolic pressure and lower arterial compliance initially seen. Elevated concentrations of inflammatory markers are generally the result of infection, whether it is a simple acute cold or flu or because of more severe chronic conditions such as tuberculosis and HIV [21], [22]. In this study population, the levels of both NT-proBNP and inflammatory markers were not abnormally elevated. Yet the associations obtained in this study suggest that even low concentrations of inflammatory markers may contribute to elevated risk of cardiovascular damage and possible future cardiac events in the black male group. Conversely, white men exhibited higher levels of lipids and had higher mean BMI, but the lack of association between NT-proBNP and inflammatory markers, might suggest that some intrinsic cardioprotective mechanism(s) may be at work or early onset alterations are not prevalent and warrant further investigation.

The underlying and combined mechanisms relating NT-proBNP to CRP and suPAR in cardiovascular disease are not well understood and we can therefore only speculate to explain our findings. A cascade of contributing factors is involved in the poor cardiovascular state of the black South African population. Among these factors are adverse lifestyle choices which include alcohol misuse, insufficient dietary intake and excessive smoking. In turn, these factors contribute to alterations in hemodynamic and metabolic processes after which systemic inflammation becomes unavoidable [23], [24], [25]. This is in line with the known risk of cardiovascular disease among black South Africans [26]. With the development of systolic and diastolic dysfunction, due to obesity, lifestyle or predisposition, the volume load of the ventricles increases. This elevation in volume load results in cardiac myocyte stretch and stimulates the release of NT-proBNP [27], [28], [29].

Normally, the natriuretic peptide clearance receptor type C and the kidneys maintain the concentrations of BNP and its amino fragment to restore the equilibrium of the natriuretic peptide system and its effects [30]. However, once the heart is subjected to chronic myocyte stretch because of multiple components escalating in volume overload and augmented NT-proBNP concentrations, CRP and suPAR release are augmented by the expression of interleukin-6. The urokinase plasminogen activator will bind to its receptor (uPAR) to convert plasminogen into active plasmin [31]. Plasmin, in turn, has a direct effect on extracellular matrix degradation and can also convert pro-matrix metalloproteinases into active matrix metalloproteinases [32]. Metalloproteinases contribute to extracellular matrix degradation and ultimately to invasion of pro-inflammatory components [31]. As a result of either a systemic inflammatory or acute phase response, or also extracellular matrix remodelling, the heart is subjected to ventricular hypertrophy due to increased volume overload [33].

Our study has certain limitations. It was cross-sectional and therefore causality cannot be inferred due to confounding variables or unknown factors that were associated with both NT-proBNP and inflammation. The black men in this study were from a low socio-economic class (although all participant were from the same geographical region), which could have rendered these subjects as high risk for developing disease, due to limited healthcare provision. However, no interactions were observed with socio-economic standing. NT-proBNP is known as a stable and sensitive marker of cardiac function, including right ventricular dysfunction [34], left ventricular systolic dysfunction, and early cardiac alterations [35]. NT-proBNP was therefore used only as a marker of potential cardiac damage. Echocardiography data was not available for this study. However, this was a well-designed study supervised under controlled conditions, and contributes to the limited literature available on NT-proBNP and its associations with inflammatory markers in a South African population. Furthermore, this result is clinically relevant and suggests that if intervention does not substantiate the risk observed in this black population, in due time they may be more prone to develop early subclinical cardiovascular damage and potential detrimental cardiac events.

In conclusion, NT-proBNP persistently associated with CRP and suPAR in black men, but not in white men. Our result suggests that a low-grade inflammatory state may potentiate adverse changes in hemodynamic strain on the heart. This is in line with the higher prevalence of hypertension and early onset hemodynamic alterations known to this group of black men. However, these findings need confirmation in larger prospective and experimental studies to identify potential mechanisms of early onset cardiovascular changes.

## References

[1] Kengne AP, Anderson CS (2006) The neglected burden of stroke in Sub-Saharan Africa. Int J Stroke 1 4: 180–190. 18706015 10.1111/j.1747-4949.2006.00064.x

[2] Fourie CMT, Van Rooyen JM, Kruger A, Olsen MH, Eugen-Olsen J, et al (2012) Soluble urokinase plasminogen activator receptor (suPAR) is associated with metabolic changes in HIV-1-infected Africans: a prospective study. Inflammation 35 1: 221–229. 21384094 10.1007/s10753-011-9308-6

[3] Gottdiener JS, Arnold AM, Aurigemma GP, Polak JF, Tracy RP, et al (2000) Predictors of congestive heart failure in the elderly: the Cardiovascular Health Study. J Am Coll Cardiol 35: 1628–1637. 10807470 10.1016/s0735-1097(00)00582-9

[4] Yu H, Rifai N (2000) High-sensitivity C-reactive protein and atherosclerosis: from theory to therapy. Clin Biochem 33: 601–610. 11166006 10.1016/s0009-9120(00)00186-7

[5] Ridker PM, Stampfer MJ, Rifai N (2001) Novel risk factors for systemic atherosclerosis. JAMA 285: 2481–2485. 11368701 10.1001/jama.285.19.2481

[6] Meigs JB, Hu FB, Rifai N, Manson JAE (2004) Biomarkers of endothelial dysfunction and risk of type 2 diabetes mellitus. JAMA 291: 1978–1986. 15113816 10.1001/jama.291.16.1978

[7] Eugen-Olsen J, Andersen O, Linneberg A, Ladelund S, Hansen T, et al (2010) Circulating soluble urokinase plasminogen activator receptor predicts cancer, cardiovascular disease, diabetes and mortality in the general population. J Intern Med 268: 296–308. 20561148 10.1111/j.1365-2796.2010.02252.x

[8] Sehestedt T, Lyngbæk S, Eugen-Olsen J, Jeppesen J, Andersen O, et al (2011) Soluble urokinase plasminogen activator receptor is associated with subclinical organ damage and cardiovascular events. Atherosclerosis 216: 237–243. 21354571 10.1016/j.atherosclerosis.2011.01.049

[9] Nielsen SE, Schjoedt KJ, Rossing K, Persson F, Schalkwijk CG, et al (2012) Levels of NT-proBNP, markers of low-grade inflammation, and endothelial dysfunction during spironolactone treatment in patients with diabetic kidney disease. J RAAS doi:10.1177/1470320312460290. 10.1177/1470320312460290 23108194

[10] McCullough PA, Sandberg KR (2003) B-type natriuretic peptide and renal disease. Heart Fail Rev 8: 355–358. 14574057 10.1023/a:1026195332025

[11] Jensen J, Ma L, Fu MLX, Svaninger D, Lundberg P, et al (2010) Inflammation increases NT-proBNP and the NT-proBNP/BNP ratio. Clin Res Cardiol 99: 445–452. 20229122 10.1007/s00392-010-0140-z

[12] O'Brien E, Asmar R, Beilin L, Imai Y, Mancia G, et al (2005) Practice guidelines of the European Society of Hypertension for clinic, ambulatory and self blood pressure measurement. J Hypertens 23: 697–701. 15775768 10.1097/01.hjh.0000163132.84890.c4

[13] Imholz BPM, Wieling W, Van Montfrans GA, Wesseling KH (1998) Fifteen years' experience with finger arterial pressure monitoring. Cardiovasc Res 38: 605–616. 9747429 10.1016/s0008-6363(98)00067-4

[14] Guelen I, Westerhof BE, Van der Sar GL, Van Montfrans GA, Kiemeneij F, et al (2008) Validation of brachial artery pressure reconstruction from finger arterial pressure. J Hypertens 26: 1321–1327. 18551006 10.1097/HJH.0b013e3282fe1d28

[15] Marfell-Jones M, Olds T, Stewart A, Carter L (2006) International standards for anthropometric assessment. *ISAK: Potchefstroom, South Africa*; 32–89 p.

[16] Tamakoshi K, Yatsuya H, Kondo T, Hori Y, Ishikawa M, et al (2003) The metabolic syndrome is associated with elevated circulating C-reactive protein in healthy reference range, a systemic low-grade inflammatory state. Int J Obes 27: 443–449. 10.1038/sj.ijo.0802260 12664077

[17] Ossowski L, Aguirre-Ghiso JA (2000) Urokinase receptor and integrin partnership: coordination of signaling for cell adhesion, migration and growth. Curr Opin Cell Biol 12: 613–620. 10978898 10.1016/s0955-0674(00)00140-x

[18] Morrow DA, Braunwald E (2003) Future of biomarkers in acute coronary syndromes: moving toward a multimarker strategy. Circulation 108: 250–252. 12876133 10.1161/01.CIR.0000078080.37974.D2

[19] Pepys BM (1983) Acute phase proteins with special reference to C-reactive protein and related proteins (pentaxins) and serum amyloid A protein. Adv Immunol 34: 141–212. 6356809 10.1016/s0065-2776(08)60379-x

[20] Thunø M, Macho B, Eugen-Olsen J (2009) suPAR: the molecular crystal ball. Dis Markers 27: 157–172. 19893210 10.3233/DMA-2009-0657 PMC3835059

[21] McDade TW (2003) Life history theory and the immune system: steps toward a human ecological immunology. Am J Phys Anthropol 122 37: 100–125. 10.1002/ajpa.10398 14666535

[22] Crimmins EM, Finch CE (2006) Infection, inflammation, height, and longevity. Proc Natl Acad Sci USA 103: 498–503. 16387863 10.1073/pnas.0501470103 PMC1326149

[23] Hansson GK, Libby P (2006) The immune response in atherosclerosis: a double-edged sword. Nat Rev Immunol 6: 508–519. 16778830 10.1038/nri1882

[24] Hunt SA, Abraham WT, Chin MH, Feldman AM, Francis GS, et al (2009) ACCF/AHA Practice Guideline: Focused Update. Circulation 119: 1977–2016. 19324967 10.1161/CIRCULATIONAHA.109.192064

[25] Naghavi M, Libby P, Falk E, Casscells SW, Litovsky S, et al (2003) From vulnerable plaque to vulnerable patient: a call for new definitions and risk assessment strategies: Part I. Circulation 108: 1664–1672. 14530185 10.1161/01.CIR.0000087480.94275.97

[26] Opie LH, Seedat YK (2005) Hypertension in sub-Saharan African populations. Circulation 112: 3562–3568. 16330697 10.1161/CIRCULATIONAHA.105.539569

[27] Must A, Spadano J, Coakley EH, Field AE, Colditz G, et al (1999) The disease burden associated with overweight and obesity. JAMA 282: 1523–1529. 10546691 10.1001/jama.282.16.1523

[28] Raizada V, Thakore K, Luo W, McGuire P (2001) Cardiac chamber-specific alterations of ANP and BNP expression with advancing age and with systemic hypertension. Mol Cell Biochem 216: 137–140. 11216858 10.1023/a:1011027231702

[29] Uusimaa P, Tokola H, Ylitalo A, Vuolteenaho O, Ruskoaho H, et al (2004) Plasma B-type natriuretic peptide reflects left ventricular hypertrophy and diastolic function in hypertension. Int J Cardiol 97: 251–256. 15458692 10.1016/j.ijcard.2003.10.015

[30] Hall C (2005) NT-ProBNP: the mechanism behind the marker. J Card Fail 11: S81–83. 15948107 10.1016/j.cardfail.2005.04.019

[31] Binder BM, Mihaly J, Prager GW (2007) uPAR-uPA-PAI-1 interactions and signaling: a vascular biologist's view. Thromb Haem 97: 336–342. 17334498

[32] Prager GW, Breuss JM, Steurer S, Mihaly J, Binder BR (2004) Vascular endothelial growth factor (VEGF) induces rapid prourokinase (pro-uPA) activation on the surface of endothelial cells. Blood 103: 955–962. 14525763 10.1182/blood-2003-07-2214

[33] Sackner-Bernstein JD (2000) The myocardial matrix and the development and progression of ventricular remodeling. Curr Cardiol Rep 2: 112–119. 10980881 10.1007/s11886-000-0007-4

[34] Yap LB (2004) B-type natriuretic peptide and the right heart. Heart Fail Rev 9: 99–105. 15516857 10.1023/B:HREV.0000046364.68371.b0

[35] Pemberton CJ, Johnson ML, Yandle TG, Espiner EA (2000) Deconvolution analysis of cardiac natriuretic peptides during acute volume overload. Hypertension 36: 355–359. 10988264 10.1161/01.hyp.36.3.355

